# Uniaxial Static Strain Promotes Osteoblast Proliferation and Bone Matrix Formation in Distraction Osteogenesis In Vitro

**DOI:** 10.1155/2020/3906426

**Published:** 2020-08-12

**Authors:** Zhengqiang Li, Junfa Zheng, Di Wan, Xiaoqin Yang

**Affiliations:** ^1^Department of Oral and Maxillofacial Surgery, Stomatological Hospital, Southern Medical University, Guangzhou 510280, China; ^2^Department of Oral and Maxillofacial Surgery, School of Stomatology, Jilin University, Changchun 130021, China

## Abstract

**Objective:**

We aimed at investigating the effects of uniaxial static strain on osteoblasts in distraction osteogenesis (DO).

**Methods:**

To simulate the mechanical stimulation of osteoblasts during DO, 10% uniaxial static strain was applied to osteoblasts using a homemade multiunit cell stretching and compressing device. Before and after applying strain stimulation, the morphological changes of osteoblasts were observed by inverted phase-contrast microscopy, Coomassie blue staining, and immunofluorescence. Alkaline phosphatase (ALP) activity, mRNA levels (proliferating cell nuclear antigen [PCNA], ALP, Runx2, osteocalcin [OCN], collagen type I, hypoxia-inducible factor- [HIF-] 1*α*, and vascular endothelial growth factor [VEGF]), and protein levels (Runx2, OCN, collagen type I, HIF-1*α*, and VEGF) were evaluated by using ALP kit, real-time quantitative reverse transcription-polymerase chain reaction, western blot, and enzyme-linked immunosorbent assay.

**Results:**

After the mechanical stimulation, the cytoskeleton microfilaments were rearranged, and the cell growth direction of the osteoblasts became ordered, with their direction being at an angle of about 45° from the direction of strain. The proliferation of osteoblasts and the expression levels of mRNA and protein of ALP, Runx2, OCN, collagen type I, HIF-1*α*, and VEGF were significantly higher than in the nonstretch control groups.

**Conclusion:**

Our homemade device can exert uniaxial static strain and promote the proliferation of osteoblasts and bone matrix formation. It can be used to simulate the mechanical stimulation of osteoblasts during DO.

## 1. Introduction

Distraction osteogenesis (DO), known as “endogenous bone tissue engineering,” is a predictable and effective surgical technique to regenerate new bone in the gap between two bone segments to lengthen or widen the bone [1, 2]. This technology was first reported by Italian scholar Codivilla in 1905 to treat a shortened femur [3], and then it was systematically developed for orthopedic surgery by Dr. Gabriel A. Ilizarov in the 1950s [4–6]. After many craniomaxillofacial animal models of DO were established, DO technology has gradually become a research hotspot in oral and maxillofacial surgery and plastic surgery [7, 8]. Today, DO is being widely used to treat craniomaxillofacial congenital malformations or acquired large bone defects due to trauma, infections, and postresection of tumors.

**Figure 1:**
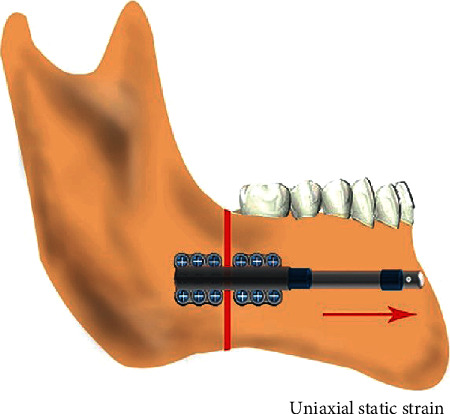
A sketch of distraction osteogenesis in vivo, showing the direction of strain (red arrow).

The DO procedure requires the application of a gradual and controlled mechanical distraction force to the bone segments, which is crucial for new bone formation. However, the molecular mechanisms of DO remain unknown. Moreover, complications such as premature consolidation and incomplete callus formation [9] during DO still exist. All these questions have limited the application of DO in clinics. Therefore, there is a burning need to understand the underlying specific molecular mechanisms of DO, which can help us better regulate successful bone formation and reduce the complications during the DO process. Many previous studies have attempted to elucidate the mechanisms of DO, and various cell mechanical loading devices (such as FlexCell system [10] and four-point bending [11]) have been developed to simulate the mechanical environment of osteoblasts during DO. However, these studies were severely limited because the forces applied to osteoblasts were mainly fluid shear stress, equiaxial strain, multiaxial strain, centrifugal force, and cyclic reciprocating strain. These kinds of forces did not replicate the actual mechanical environment of osteoblasts during DO [12, 13]. Actually, the mechanical stimulation of osteoblasts in DO involves mainly uniaxial static strain along the stretching direction [14, 15] (Figure 1).

**Figure 2:**
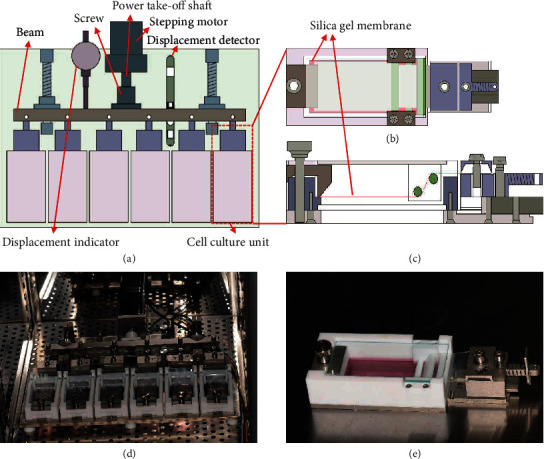
Schematic diagrams of (a) the homemade multiunit cell stretching and compressing device; (b) top view; and (c) side view of the cell culture unit; photographs of the (d) multiunit cell stretching and compressing device; and (e) the cell culture unit.

Our research group has successfully developed a multiunit cell stretching and compressing device (the patent number of invention: ZL200910164248, China) (Figure 2), which can exert uniaxial static strain to simulate the mechanical environment of DO. The mechanical stimulation can be transmitted to the cultured cells through the deformation of a silica gel membrane with good biocompatibility. Accordingly, 1%, 5%, 10%, and 15% uniaxial static strains were applied on the cultured osteoblasts by the device. The Cell Counting Kit-8 (CCK-8; Dojindo Laboratories, Kumamoto, Japan) assay showed that 10% uniaxial static strain is best able to promote the proliferation of osteoblasts. To further elucidate the underlying molecular mechanisms of new bone formation during the process of DO, 10% uniaxial static strain was applied on the osteoblasts by the device. The morphology changes, proliferation, and the synthesis and secretion of bone matrix by the osteoblasts were evaluated through real-time polymerase chain reaction (PCR), western blot, and enzyme-linked immunosorbent assay (ELISA).

## 2. Materials and Methods

### 2.1. Osteoblast Isolation and Culture

The Animal Care and Use Committee of Southern Medical University approved the procedures of the study. Osteoblasts were isolated and cultured in accordance with a previously published reference [16]. Briefly, the skull was isolated from 1- to 2 d old neonatal Sprague Dawley (SD) rats under aseptic conditions and then minced into fragments of about 0.5 mm^3^. The bone fragments were washed with phosphate-buffered saline (PBS) (containing 100 units/mL penicillin and 100 mg/mL streptomycin) three times and digested with 0.25% trypsin (Gibco, Life Technologies, Carlsbad, CA, USA) at 37°C for 30 min with shaking. Then, they were washed with serum-free Dulbecco's modified Eagle's medium (DMEM; Sigma-Aldrich, St. Louis, MO, USA) three times after the trypsin solution was removed. A total of 0.1% collagenase II (Gibco) in serum-free DMEM was added to the bone fragment, allowing digestion at 37°C for 30 min with shaking. The supernatant was centrifuged at 1,000 rpm for 5 min to obtain the cell pellet. The pellet was resuspended in serum-free DMEM and centrifuged; this process was repeated twice. Finally, the pellet was resuspended in DMEM supplemented with 10% fetal bovine serum (FBS; Gibco), 100 units/mL penicillin, and 100 mg/mL streptomycin and then seeded in a 25 cm^2^ culture flask at a density of 2 × 10^5^ cells/mL. After incubating for 10 min at 37°C and 5% CO_2_ (Thermo Scientific 311; Marietta, GA, USA), the culture medium and suspended osteoblasts were transferred to a new 25 cm^2^ culture flask to remove the earlier adherent fibroblasts. Finally, the purified osteoblasts were incubated at 37°C and 5% CO_2_. The culture medium was refreshed every 2–3 d. The morphology and growth of the cells were observed and photographed using an inverted phase-contrast microscope. The cells were subcultured at a ratio of 1 : 3 upon reaching 80% confluence, and cells at the third passage were used for the following experiments.

### 2.2. Cell Proliferation Activity under Different Uniaxial Static Strains

The schematic diagram and photograph of the device are shown in Figure 2. A screw connects the power take-off shaft of the stepping motor and the beam, and the beam is connected with the six cell culture units. The uniaxial static strain exerted by the stepping motor is transmitted to the silica gel membrane in the cell culture units and causes cell deformation. The displacement detector detects and controls the tensile and compression displacement, and the displacement indicator displays the displacement.

For our study, the osteoblast suspension (2 mL) was seeded on a silica gel membrane (cell seeding density: 2 × 10^4^ cells/mL) coated with collagen type I from rat tail (Sigma-Aldrich) in a cell culture unit and further incubated for 24 h at 37°C and 5% CO_2_, allowing for osteoblast attachment. Then, an additional 10 mL of DMEM supplemented with 10% FBS was replenished. Subsequently, 1%, 5%, 10%, and 15% uniaxial static strains were applied to the osteoblasts 24 h later using the multiunit cell stretching and compressing device. The culture medium was refreshed every 2–3 d. Control groups (nonstretch groups) were cultured under the same conditions, except for the application of mechanical stimulation. The proliferation activity of cells was analyzed using a CCK-8 (Dojindo Laboratories,) assay at days 1, 3, 5, and 7 of the experiment. Briefly, the adherent osteoblasts of the stretch and nonstretch groups were collected from the silica gel and transferred to a culture plate. CCK-8 solution was added to each well (*n* = 4). The cells were then incubated at 37°C and 5% CO2 for 2 h. Optical density was measured at 450 nm using a multiscan spectrometer (Varioskan Flash; Thermo Electron Corporation). Each experiment was repeated three times independently.

### 2.3. Osteoblast Morphology

According to the CCK-8 assay, 10% uniaxial static strain is best able to promote the proliferation of osteoblasts. Therefore, in the following experiments, we used the homemade device to apply a 10% uniaxial static strain to simulate the mechanical environment of osteoblasts during DO in vivo. Inverted phase-contrast microscopy, Coomassie blue staining, and immunofluorescence were used to observe the changes in morphology and growth direction of osteoblasts. After culturing for 3 d, the osteoblasts of the stretch and nonstretch groups were washed with PBS and fixed with 4% paraformaldehyde for 20 min. Next, they were rinsed three times with deionized water and stained with Coomassie bright blue staining solution (2 g/L) (Sigma-Aldrich) for 60 min at room temperature. The cells were washed three times with deionized water, and methanol–glacial acetic solution was added to stop the staining reaction until the cytoplasm was slightly clear under an inverted phase-contrast microscope. Finally, the osteoblast morphology was observed and photographed with an inverted phase-contrast microscopy.

The immunofluorescence procedure was as follows. First, after culturing for 3 d, the osteoblasts of the stretch and nonstretch groups were washed with PBS and fixed with 3.7% paraformaldehyde in PBS for 30 min at room temperature. Next, the cells were rinsed three times with 0.1% Triton X-100 (Sigma-Aldrich) in PBS. Thereafter, 1 mL of diluted (1 : 50) Actin-Tracker Green solution (Sigma-Aldrich) was added to the osteoblasts on the silica gel membrane and incubated for 40 min at room temperature in the dark. The osteoblasts were washed and 1 mL of 4′, 6-diamidino-2-phenylindole (DAPI) staining solution (Sigma-Aldrich) was added to cover the osteoblasts and maintained for 5 min at room temperature. After aspirating the DAPI staining solution and washing, the osteoblasts were observed and photographed under a fluorescence microscope.

### 2.4. ALP Activity Assay

The ALP activity of osteoblasts was detected using an ALP kit (Nanjing Jiancheng Bioengineering Institute, Jiangsu, China) after culturing for 1, 3, and 5 d. Briefly, the adherent osteoblasts of the stretch and nonstretch groups were separated and collected from the silica gel by trypsin digestion (2,000 rpm for 1 min). An aliquot (100 *μ*L) of cell lysate containing 0.05% Triton X-100 was used to lyse the osteoblasts at 4°C for 12 h. A 20 *μ*L sample and 100 *μ*L of substrate buffer (containing *p*-nitrophenyl phosphate) were added to a 96-well culture plate, shaken for 1 min, and incubated at 37°C for 15 min, followed by addition of 80 *μ*L of reaction termination liquid and shaking for 1 min. Finally, the absorbance value was measured at 520 nm wavelength, and the resulting nitrophenol level was calculated following the instructions in the ALP kit. Referring to the normal standard, total intracellular protein content was detected using the bicinchoninic acid (BCA) Protein Assay Kit (Nanjing Jiancheng Bioengineering Institute, Jiangsu, China). The relative ALP activity was calculated by dividing the amount of nitrophenol by the corresponding total protein.

### 2.5. Quantitative Real-Time Reverse Transcription-PCR (RT-PCR)

RT-PCR was performed to evaluate the expression levels of proliferating cell nuclear antigen (PCNA), ALP, Runx2, osteocalcin (OCN), collagen type I, hypoxia-inducible factor- (HIF-) 1*α*, and vascular endothelial growth factor (VEGF). Briefly, after culturing for 1, 3, and 5 d, the silica gel membranes with the attached osteoblasts from the stretch and nonstretch groups were transferred to a Petri dish, and the total mRNA was extracted using TRIzol reagent (Invitrogen, Life Technologies, Carlsbad, CA, USA).

Reverse transcription was performed to synthesize cDNA from the purified RNA using Oligo (dT) primers (Promega, San Luis Obispo, CA, USA) and SuperScript III reverse transcriptase (Invitrogen). Finally, cDNA was subjected to real-time PCR (Applied Biosystems 7300; Applied Biosystems Foster City, CA, USA) using the SYBR Green probe (PerfeCTa SYBR Green FastMix, ROX; Quanta Biosciences, Gaithersburg, MD, USA) with custom-designed primers (Takara Bio, Dalian, China; Table 1).

Expression of the target gene was first normalized to that of the housekeeping gene glyceraldehyde 3-phosphate dehydrogenase (GAPDH) in the same sample (△Ct = threshold cycle change). Then, the target gene of the experimental groups was normalized to the average baseline expression of the gene measured for the control groups (*△△*Ct). The 2^–*△△*Ct^ method was used to convert the normalized gene expression levels in accordance with a previously published reference [17].

### 2.6. Western Blot

Western blot was performed to evaluate the protein levels expressed by the osteoblasts, including Runx2, collagen type I, HIF-1*α*, and GAPDH. Briefly, the adherent osteoblasts were scraped from the silica gel membrane and centrifuged at 2,000 rpm for 1 min. After lysing the cells in lysis buffer for 15 min on ice, cell debris was removed by centrifugation at 14,000 rpm for 15 min at 4°C. The supernatant was collected and stored at –80°C until use. The protein content was determined using the Bradford method, and the samples were diluted to 1 *μ*g/*μ*L with the lysate. Equivalent amounts of protein were separated with 5% stacking gel and 10% separating gel by sodium dodecyl sulfate-polyacrylamide gel electrophoresis (SDS-PAGE) and then transferred onto polyvinylidene fluoride (PVDF) membranes (Millipore, Billerica, MA, USA) for 90 min at 120 V using a Trans-Blot SD electrophoresis apparatus (Bio-Rad). The PVDF membranes were blocked with 5% skimmed milk for 1 h at room temperature and incubated with primary antibodies (including Runx2, collagen type I, HIF-1*α*, and GAPDH from mouse) (R&D Systems, Minneapolis, MN, USA) at 4°C overnight, then washed three times with 0.1% Tween–Tris-buffered saline (TBS) (TTBS, pH 7.5), followed by incubation with horseradish peroxidase-conjugated secondary antibodies (1 : 2,000) (R&D Systems) for 1 h at 37°C. Finally, the specific protein bands were visualized with an Immuno-Star Western C kit on a ChemiDoc XRS+ System (Bio-Rad) using BCA as a standard.

### 2.7. ELISA Tests

Protein levels of OCN and VEGF secreted by the osteoblasts were analyzed using an ELISA kit (R&D Systems). Briefly, after culturing for 1, 3, and 5 d, the culture supernatant of the stretch and nonstretch groups was collected, centrifuged at 3,000 rpm for 20 min at 4°C, and then stored at –20°C for use. First, we added 50 *μ*L sample dilutions at a dilution ratio of 1 : 5 to the wells. The plates were sealed and incubated at 37°C for 30 min. Then, we aspirated the liquid and washed the wells. Finally, 50 *μ*L of enzyme-labeled reagent, 50 *μ*L of chromogenic reagent A, and 50 *μ*L of chromogenic reagent B were added to the wells, and the plates were incubated at 37°C for 15 min in the dark; later, 50 *μ*L of stop solution was added to each well to stop the reaction. Zeroing was performed in a blank well. The absorbance of each sample was detected using an immunoenzyme analyzer (Thermo Scientific) at 450 nm. Protein concentrations were calculated using a standard curve according to instructions.

### 2.8. Statistical Analysis

All quantitative values are presented as mean ± standard deviation. Statistical analyses were performed using the Statistical Package for Social Sciences (SPSS) version 19.0 software (IBM Corp., Armonk, NY, USA). Differences were assessed by one-way analysis of variance (ANOVA) and were considered statistically significant at *P* < 0.05. The data are indicated with ^∗^ for *P* < 0.05. All the experiments were repeated at least three times.

## 3. Results

### 3.1. CCK-8 Assay

**Figure 3:**
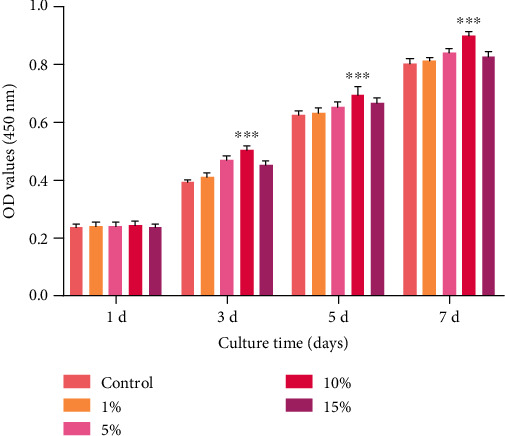
CCK-8 assay after application of 0% (control), 1%, 5%, 10%, and 15% uniaxial static strain to osteoblasts using the device. ^∗∗∗^*P* < 0.001 when compared with other groups.

As shown in Figure 3, the osteoblasts in the five groups kept growing during the experiment. However, osteoblasts grew the fastest under 10% uniaxial static strain at 3, 5, and 7 d when compared to other groups.

### 3.2. In Vitro Culture and Morphological Observation of Osteoblasts

**Figure 4:**
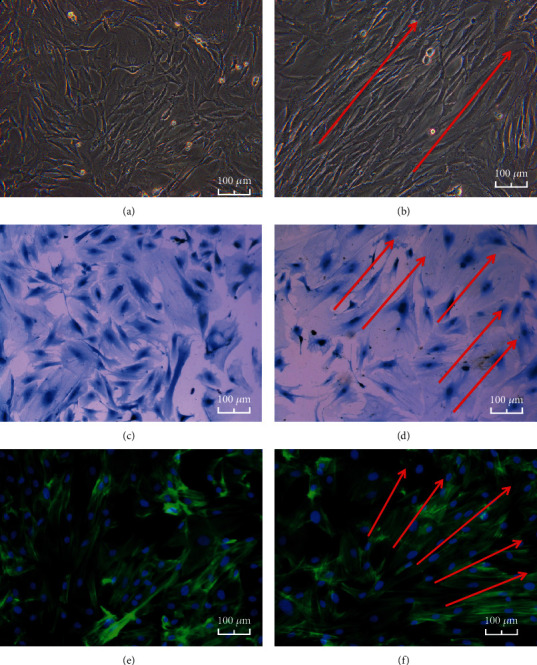
Osteoblast morphology of (a, c, and e) the control group and (b, d, and f) the experimental group when observed as follows: (a, b) under an inverted phase-contrast microscope; (c, d) after Coomassie blue staining; and (e, f) by immunofluorescence. The orientations of the osteoblasts (red row).

The osteoblasts of the nonstretch control groups were mostly short fusiform, triangular, or star-shaped and in a disorderly array (Figures 4(a), 4(c), and 4(d)). After being subjected to 10% uniaxial static strain for 3 d, the osteoblasts became elongated and appeared as long spindles under the inverted phase-contrast microscope. The growth direction gradually developed from random disorder to cells aligned in the same direction, at an angle of about 45° from the direction of strain (Figure 4(b)). Coomassie blue staining and immunofluorescence staining images showed that the osteoblasts were still slightly randomly oriented (Figures 4(d) and 4(f)). The orientation of osteoblasts in images D and F was not so regular as that in image B. However, the orientations of the osteoblasts had a trend to arrange regularly in images D and F.

### 3.3. ALP Activity

**Figure 5:**
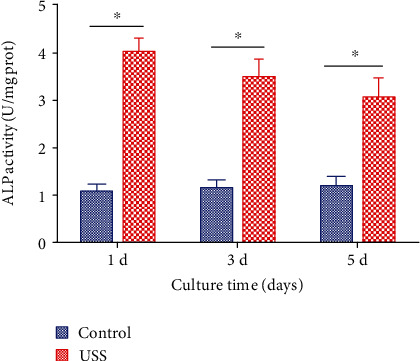
ALP activity of the control group and 10% uniaxial static strain (USS) group; *n* = 3 from one experiment; similar trends were observed in three independent experiments. ^∗^*P* < 0.05.

As a key enzyme related to bone metabolism, ALP plays an important role in bone formation and mineralization. The changes in ALP activity can reflect the differentiation ability of osteoblasts. The results (Figure 5) show that after the application of 10% uniaxial static strain, the ALP activity was higher than the control group at the three time points. The ALP activity of the experimental group was the highest on Day 1 and then gradually slowed at Days 3 and 5, while, for the control group, the activity was almost the same. The results indicated that 10% uniaxial static strain promoted the secretion of ALP by osteoblasts.

### 3.4. RT-PCR Assay

**Figure 6:**
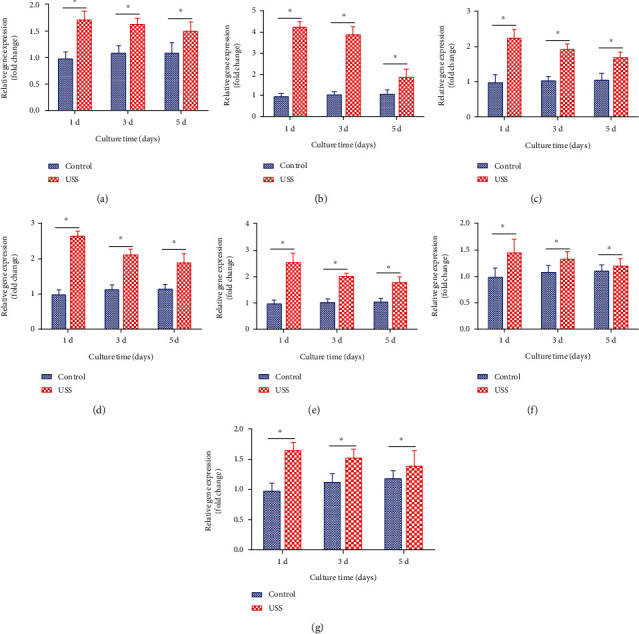
Quantitative gene expression results of (a) PCNA, (b) ALP, (c) Runx2, (d) OCN, (e) collagen type I, (f) HIF-1*α*, and (g) VEGF for osteoblasts of the 10% uniaxial static strain (USS) group and control group at Day 1, Day 3, and Day 5. Data are presented as fold changes after normalization to GAPDH. Fold changes are shown as mean ± standard deviation for *n* = 3 from one experiment; similar trends were observed in three independent experiments. ^∗^*P* < 0.05.

The expression levels of PCNA, ALP, Runx2, OCN, collagen type I, HIF-1*α*, and VEGF of osteoblasts from the experimental and control groups were measured at Days 1, 3, and 5 (Figure 6).

The expression levels of PCNA of the experimental group were the highest at Day 1 and then decreased gradually, while in the control group, the level slowly increased. Moreover, the experimental group showed significantly higher levels at all three time points compared with the control group. This suggested that 10% uniaxial static strain was able to promote the proliferation of osteoblasts.

ALP, Runx2, OCN, and collagen type I are important osteogenesis-related markers. HIF-1*α* and VEGF are important angiopoiesis-related factors. The results of mRNA expression of ALP were inconsistent with the ALP activity detected by the ALP kit. Specifically, after the application of 10% uniaxial static strain, the mRNA expression levels of ALP in the osteoblasts were higher than in the control group at Days 1, 3, and 5. The highest level was on Day 1 and it gradually decreased at Days 3 and 5, while the results for the control group were almost unchanged at the three time points.

Similarly, the mRNA levels of Runx2, OCN, collagen type I, HIF-1*α*, and VEGF showed a significantly higher expression after the application of 10% uniaxial static strain at Days 1, 3, and 5 than those of the control group. The mRNA levels reached the highest value on Day 1 and decreased gradually, while those of the control group slowly increased from Day 1 to Day 5.

### 3.5. Western Blot

**Figure 7:**
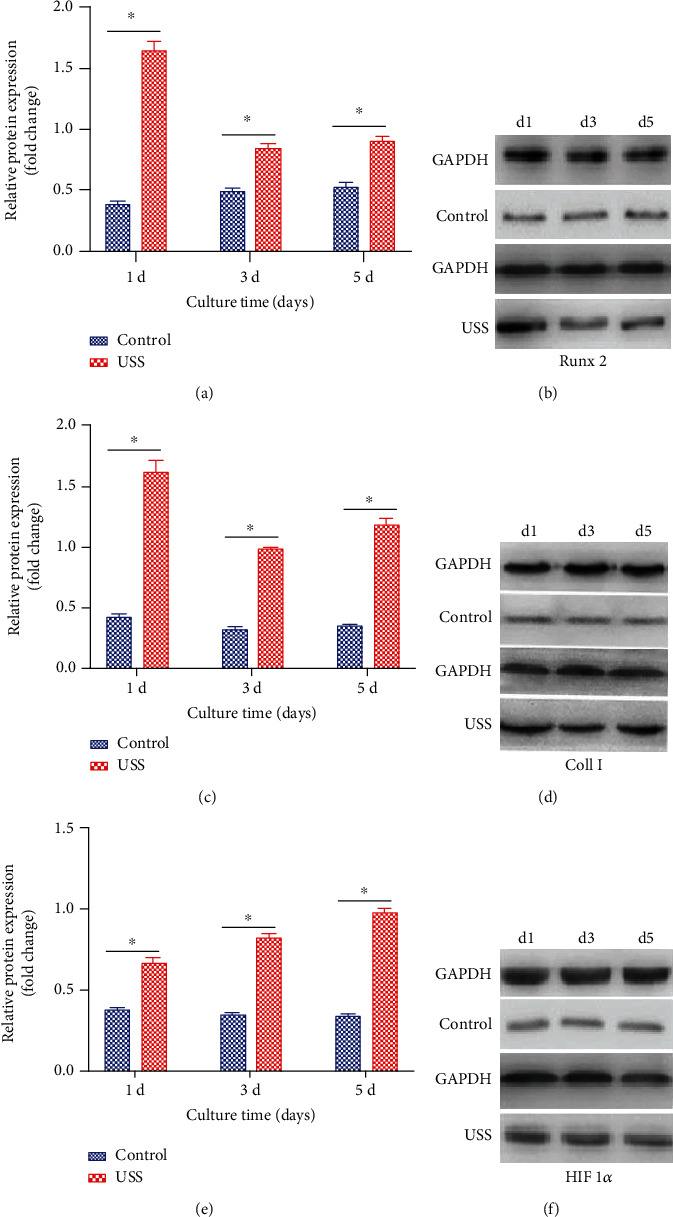
Western blot results of (a, b) Runx2, (c, d) collagen type I, and (e, f) HIF-1*α* for osteoblasts before and after application of 10% uniaxial static strain for 1, 3, and 5 d. Data are presented as fold changes after normalization to GAPDH. Fold changes are shown as mean ± standard deviation for *n* = 3 from one experiment; similar trends were observed in three independent experiments. ^∗^*P* < 0.05.

The expression amounts of the proteins Runx2, collagen type I, and HIF-1*α* were measured at Days 1, 3, and 5 (Figure 7). The amounts of protein of Runx2 and collagen type I peaked at Day 1 and decreased gradually, while the amount of protein of HIF 1*α* increased gradually and peaked at Day 5. The variation trends of Runx2 and collagen type I were consistent with the results of the mRNA expression levels detected by RT-PCR, while the variation trend of HIF 1*α* was opposite to the result of RT-PCR.

### 3.6. ELISA Tests

**Figure 8:**
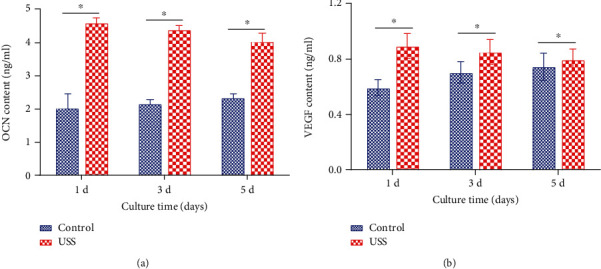
ELISA results of (a) OCN and (b) VEGF for osteoblasts of the 10% uniaxial static strain (USS) group and control group at Day 1, Day 3, and Day 5, *n* = 3 from one experiment; similar trends were observed in three independent experiments. ^∗^*P* < 0.05.

As shown in Figure 8, the expression levels of OCN and VEGF were measured at 1, 3, and 5 d after the application of 10% uniaxial static strain. The results of the experimental group decreased slightly, while that of the control group slowly increased from Day 1 to Day 5. The amounts of OCN and VEGF proteins in the experimental groups were significantly higher than those in the control groups. The variation trends were consistent with the results of mRNA expression levels detected by RT-PCR.

## 4. Discussion

Biological living tissues have potential biological plasticity. Slow and sustained mechanical force can stimulate cell proliferation, increase biosynthetic function, and promote tissue metabolism, thus leading to tissue regeneration. As Ilizarov proposed in the “law of tension-stress” of DO, gradual and continuous distraction can stimulate and maintain bone regeneration and growth [18]. However, the underlying specific molecular mechanisms of DO have not been fully understood. To better understand the mechanisms of DO, the first thing to do is create a similar mechanical microenvironment with DO in vivo. Therefore, our team developed a multiunit cell stretching device. Compared to previous studies, our experimental results demonstrate that this device can exert uniaxial static strain on osteoblasts, which can be used to better simulate the actual mechanical stimulation of osteoblasts in the DO process.

Appropriate mechanical stimulation is particularly important and can promote osteoblast growth and maintenance of function, whereas excessive or inadequate force will have no effect or cause damage to the structure and function of osteoblasts. In this study, we found that a 10% uniaxial static strain is the best for the proliferation of osteoblasts. Our result is similar to or a little different from those in other studies [19, 20]. Bhatt et al. [21] found that a 9% strain is the best for osteoblast proliferation using a Flexcell system, while Gao et al. [22] found that 12% cyclic stretch is best for osteogenesis-related gene expression in MG-63 cells. The differences may be that other factors, such as the type of strain, magnitude, frequency, and duration, may influence the mechanical response of osteoblasts.

Osteoblast is a kind of mechanosensory cell; it has a variety of mechanoreceptors, such as the extracellular matrix (ECM) and integrin, focal adhesions, and cytoskeleton, special structures of cell membranes, cadherin and ephrin, primary cilia, ion channels, and connexins [23, 24]. When subjected to a mechanical force, the mechanoreceptors of osteoblasts can feel the stimulation signal quickly, causing changes in the osteoblast skeleton, such as microfilament. In our experiment, we can see that when subjected to mechanical force, the osteoblasts became longer as observed under an inverted phase-contrast microscope. The cell growth direction gradually developed from random disorder to cells aligned in the same direction, at almost 45° angle with the direction of strain. However, the orientation of osteoblasts in images D and F was not so regular as that in image B, which were still slightly randomly oriented. The reason might be that the duration of mechanical stimulation is not enough. The dyeing process of the osteoblasts may also have some influences. Wang et al. [25] applied 10% periodic strain to epithelial cells for 30 min and found that the angle between cell arrangement and the strain direction was 70°. The duration and magnitude of mechanical stimulation might have influenced the angle between cell arrangement and the strain direction. Our results indicate that osteoblasts can respond to uniaxial static strain, leading to changes in cytoskeletal microfilaments and cell growth direction. Microfilament forms a functional complex with related proteins, integrin, and ECM. It can maintain normal cell morphology and respond to external mechanical stimulation. It also participates in the transduction of multiple signals and in material transport inside and outside the cell. Wang et al. [25] demonstrated that signal transduction of mechanical stimulation relies on the integrity of the microfilaments.

Mechanical signals can be gradually transmitted to the intracellular area and nucleus; these signals mediate the cellular functions for osteoblast proliferation, which was verified by the mRNA expression level of PCNA. In this study, it was found that the proliferation of the experimental group was higher than that of the control group, and this result was in agreement with the results reported by most researchers [26, 27]. The mechanism of promotion of cell proliferation by strain may be that strain can enhance the mitosis of osteoblasts by increasing the expression of the proto-oncogene c-fos [28].

In the present study, we also observed changes in the expression levels of ALP, OCN, collagen type I, and Runx2 after application of mechanical stimuli to osteoblasts. These proteins act synergistically to regulate the normal function of osteoblasts and affect the entire process of bone metabolism. ALP and OCN are usually considered as early and late markers of bone matrix formation, respectively [29, 30]. Collagen type I is the main matrix of the bone secreted by osteoblasts. It plays an important role in the process of adhesion, proliferation, and bone matrix mineralization of osteoblasts [31]. Runx2, also known as Cbf*α*1, is an important transcription factor in osteogenic differentiation. It can bind to osteoblast-specific *cis*-acting elements and promote the transcription and translation of ALP, OCN, and collagen type I, thereby promoting the deposition of bone matrix. The variation trends in the gene expression levels were consistent with those of the protein amounts. That is, the expression levels of the genes and the proteins (ALP, Runx2, OCN, and collagen type I) were enhanced after applying 10% uniaxial static strain, but subsequent increases in expression were gradually reduced. Kaspar et al. [32] applied a 1,000 *με* (0.1%), 1 Hz periodic reciprocating tensile strain to human osteoblasts using a four-point bending device, and they found that the ALP activity and OCN secretion were significantly reduced. Kaspar et al. [32] found that 17% periodic intermittent equiaxial strain through the Flexcell system can promote the ALP activity of osteoblast-like cells (HT-3). Kanno et al. [33] found that uniaxial cyclic strain (15% tensile stress, 0.5 Hz) promotes ALP activity and levels of OCN and Runx2 of osteoblast-like MC3T3-E1cells. When Fong et al. applied 10% continuous equiaxial strain to cranial osteoblasts of neonatal rats, the expression of collagen type I increased [27]. There are different views on whether strain can promote the expression of osteogenic-related genes in osteoblasts, probably due to the differences of osteoblast sources and cell-loading devices. Furthermore, the type, magnitude, frequency, and duration of the mechanical stimulation may also influence the mechanical response of osteoblasts. The mechanism of strain-induced osteogenesis enhancement may be that the transcription factor FosB or the cell signaling pathway of Wnt/*β*-catenin and Ras–extracellular signal-regulated kinase (ERK) 1/2–mitogen-activated protein kinase (MAPK) was activated by the strain [33–35]. In previous studies, a periodic reciprocating tensile strain of 0.1% 1 Hz significantly reduced the ALP activity and OCN secretion of osteoblasts [32], probably because the mechanical stimulation was insufficient to initiate osteogenesis-related signaling pathways.

In DO, angiogenesis runs through the whole process of new bone formation [36]. Blood vessels not only act as a transport conduit system for oxygen and nutrients for the new bone but they also promote bone formation by regulating the interaction between bone-related cells and vascular endothelial cells [37–39]. Therefore, exploring the molecular mechanisms of vascularization and finding strategies and methods to accelerate and optimize the DO process are very important in expanding the clinical application of DO. Hypoxic conditions and VEGF are essential conditions for angiogenesis during bone formation. Osteoblasts can participate in the process of vascularization of bone tissue by secreting cytokines such as HIF-1*α* and VEGF [40].

HIF-1*α* is an important transcription factor that regulates oxygen homeostasis. Under hypoxic conditions, HIF-1*α* expressed in osteoblasts can relocate to the nucleus and bind to HIF-1*β* to form active HIF-1, which can bind to the hypoxia-responsive element (HRE) on the target gene, further activating hypoxia-related genes (such as VEGF and angiopoietin) and promote angiogenesis [41]. HIF-1*α* may also directly bind to the upstream site of the osteoprotegerin (*OPG*) gene to increase the gene's expression and then promote bone formation [42]. VEGF plays an important role in angiogenesis and osteogenesis. It can be regulated by many factors, such as growth factors, hormones, transcription factors, and mechanical stimulation. Hypoxia and mechanical stress can promote the expression of osteoblast VEGF as VEGF is a downstream factor of the HIF-1*α* pathway. VEGF can interact with VEGF receptors on vascular endothelial cells; this process activates tyrosine kinases and other downstream signaling molecules, induces proliferation of vascular endothelial cells, and increases capillary permeability, all of which provide the conditions for vascular endothelial cell migration and angiogenesis [43]. The neovessel not only delivers the oxygen and nutrients needed for the formation of bone tissue but vascular endothelial cells also release osteogenic factors, such as bone morphogenetic protein- (BMP) 2 or BMP-4, to accelerate osteoblast differentiation and maturity [44].

In this experiment, the expression levels of VEGF and HIF-1*α* increased after the application of 10% uniaxial static strain. The variation trend of the mRNA expression levels of VEGF was consistent with the result of the protein contents, while the variation trend of the mRNA expression levels of HIF 1*α* was opposite to the result of the protein contents. The results are consistent with those of Fong et al. [27], who found that 10% sustained equiaxial strain increased the VEGF expression level in neonatal rat cranial osteoblasts. Similar results were obtained by Kim et al. [45] who found that VEGF secretion of bone marrow stromal cells increased after application of 0.04% equiaxial static strain. Kim et al. [45] also found that the expression of HIF-1*α* was elevated when skeletal muscle was subjected to strain. Petersen et al. found that the expression of VEGF and HIF-1*α* increased in tendon fibroblasts after applying cyclic reciprocating uniaxial tension [46]. Chang et al. [47] applied isometric cyclic tension (20%, 1 Hz) to rat vascular smooth muscle cells by using negative pressure on the elastic basement membrane and found that HIF-1*α* mRNA and protein expression increased, and that this process may be regulated by the p42/p44 MAPK kinase (MAPKK) pathway. Chang et al. [47] concluded that static strain-induced VEGF expression may be observed by regulating MAPK (ERK and p38). These findings demonstrate that static strain can promote the expression levels of VEGF and HIF-1*α* in osteoblasts, which is important for providing both blood supply and nutrition for new bone formation during DO.

## 5. Conclusion

In the present study, we applied 10% uniaxial static strain to osteoblasts using a homemade multiunit cell stretching and compressing device and investigated the biological effects by observing osteoblast morphology, as well as performing ALP activity assay, RT-PCR, western blot, and ELISA. The results showed that the cytoskeleton microfilaments were rearranged and the cell growth direction of the osteoblasts became ordered. Moreover, uniaxial static strain promoted the proliferation, osteogenesis, and angiogenesis of osteoblasts. This study provides a basis for further elucidation of the molecular mechanisms of DO and provides a possible way to control the DO process in the clinic by regulating the above osteogenesis- and angiogenesis-related factors. The relationships between the above factors in related signaling pathways will be investigated in further studies to elucidate the molecular mechanisms of DO.

## Figures and Tables

**Table 1:** Forward (F) and reverse (R) primers used for quantitative RT-PCR.

Gene	GenBank no.	Primer sequence
GAPDH	XR_009170.1	F: TATGACTCTACCCACGGCAAGT
		R: ATACTCAGCACCAGCATCACC
PCNA	NM_022381.3	F: GGGTGAAGTTTTCTGCGAGTG
		R: GACAGTGGAGTGGCTTTTGTGA
ALP	NM_013059.1	F: GACCCTGCCTTACCAACTCATT
		R: GTGGAGACGCCCATACCATCT
Runx2	NM_053470.2	F: CTTCGTCAGCGTCCTATCAGTTC
		R: CAGCGTCAACACCATCATTCTG
OCN	X04141.1	F: CCTCTCTCTGCTCACTCTGCTG
		R: ACCTTACTGCCCTCCTGCTTG
Collagen type I	BC133728.1	F: TCTGACTGGAAGAGCGGAGAG
		R: GAGTGGGGAACACACAGGTCT
HIF-1*α*	NM_024359.1	F: GCATCTCCACCTTCTACCCAAGT
		R: ACTCTCTTTCCTGCTCTGTCTGG
VEGF	AY702972.1	F: CTGTACCTCCACCATGCCAAGT
		R: AGATGTCCACCAGGGTCTCAAT

## Data Availability

The data used to support the findings of this study are available from the corresponding author upon request.
